# Study on demetallization of heavy crude oil using different zeolitic materials

**DOI:** 10.1038/s41598-025-04348-y

**Published:** 2025-06-20

**Authors:** I. M. Othman, Z. F. Ghattas, S. N. Halim, M. A. Elnhal, I. H. Saleh, M. Elsafi

**Affiliations:** 1 Department of Environmental Studies, Institute of Graduate Studies and Research (IGSR), Alexandria University, Alexandria, Egypt; 2 Middle East Oil Refinery, Midor, Amreya Free Zone, Alexandria, Egypt; 3 Physics Department, Faculty of Science, Alexandria University, Alexandria, 21511 Egypt

**Keywords:** Crude oil, Demetallization process, Zeolite, Heavy metals, Nickel, Vanadium

## Abstract

Refiners downstream worldwide are devoting more attention to managing industrial impacts and environmental contamination as an approach of reducing the trace metal content of heavy crude oil and its refined products. The removal of Cr^2+^, Ni^2+^, V^4+^, and Zn^2+^ (the most significant metal ions) from Egyptian heavy crude oil at the Belayim Desert (BD) oil field has been studied in this work applying the adsorption demetallization technique. Two types of zeolitic materials have been used in the metal removal experiments. Different operating parameters such as contact time, adsorbent concentration and initial metal ions concentration (crude oil quantity) were investigated for their effects on metal removal efficiency. The experimental results of adsorption test showed that the optimal removal conditions using NZ occurred after 6 h contact time, when using 0.5 g of NZ stirred with 50 ml crude oil, these experimental conditions produced maximum V, Ni, and Cr removal efficiencies as 75.6, 73.9, and 81.8% respectively, that cleared at PXRD and EDX analysis to confirm the highly tendency of NZ to remove metal ions from BD crude oil. While the optimal removal conditions using SZ occurred after 12 h contact time, when using 2 g of SZ stirred with 50 ml crude oil, these experimental conditions produced maximum Zn and Cr removal efficiencies as 70.5, 69.17% respectively, but the maximum removal efficiency of V and Ni obtained after 26 h contact time, 2 g SZ with 50 ml crude oil reached to 68.9%. In addition to the results revealed that Natural Zeolite (NZ) is more efficient than Synthetic Zeolite (SZ) in extracting metal ions mainly Ni and V ions from BD crude oil. Also, the selectivity of NZ for heavy metals removal efficiencies are Cr > V > Ni > Zn. Whilst the selectivity of SZ for heavy metals removal efficiencies are Cr > Zn > V > Ni.

## Introduction

The need for oil and gas as a transportation fuel remains significant, even with the contribution from other fossil fuels like coal and natural gas^1^. Light oil stocks have been exhausted as a result, and oil prices have increased and become more constrained^2^. As consequence, the amount of light oil decreases while the quantity of heavy oils increases along with contaminants like metals, sulfur, asphaltenes, nitrogen, etc. These contaminants increase the cost of oil processing, which lowers the oil’s desirability. Recently, new technologies have been investigated to enhance the amount and quality of fuel for transportation derived from natural sources other than conventional one’s crude^1^. Research is currently being conducted in a number of other areas, such as the production of biofuels, the conversion of gas to liquid, and the refining of unconventional oil^1–3^. Several technologies have been researched for enhancing heavy oil output for transportation fuel^4^. The most effective of these methods is hydrocracking, which produces low selectivity to coke and high selectivity to middle distillate. The crude stock used in hydro cracking often has large concentrations of heavy oil-containing Ni and V metals, which are easily poisoned or deactivated by traditional acid catalysts^5–7^. These and other metals have made it necessary to seek for substances that can successfully extract them from crude oil during hydro-processing. Finding a way to pretreat the heavy crude oil to lower the metal ion content before it enters the downstream processing units would be a potential solution to solve this issue. Because catalysts would not become poisoned as quickly, their lifetime and throughput rate would both increase, resulting in a cost reduction.

Since zeolites have a large surface area and exceptional catalytic and ionic exchange properties, they have been used in numerous industrial applications such as water softening, heterogeneous catalysis, separation, and environmental remediation^8,9^. They can also be used to effectively remove metallic ions from crude oil. Regardless, zeolite is a type of crystalline alumino-silicate mineral that originates by synthesizing three-dimensional tetrahedral molecule configurations that are linked to O_2_ participating atoms^10^. Zeolites are undoubtedly ion exchangers and have been used as a catalyst for the catalytic cracking of heavy oils as well as an effective means of reducing or even minimizing heavy metal levels from wastewaters^11,12^. According to Benhammou et al.^13^, the following equations can be used to describe ion exchange processes that occur between exchangeable cations (Na^+^, K^+^, Ca^2+^, and Mg^2+^) found in zeolite structures and cations (Mn^+^) in the solution:

1 $${\text{Zeolite}} \equiv {\text{n}}\left( {{\text{Na}},{\text{ K}}} \right)^{ + } + {\text{Mn}}^{ + } \leftrightarrow {\text{ zeolite}} \equiv {\text{Mn}}^{ + } + {\text{n}}\left( {{\text{Na}},{\text{ K}}} \right)^{ + }$$

2 $${\text{Zeolite}} \equiv {\text{n}}\left( {{\text{Ca}},{\text{ Mg}}} \right)^{{{2} + }} + {\text{2Mn}}^{ + } \leftrightarrow {\text{zeolite}} \equiv {\text{2Mn}}^{ + } + {\text{n}}\left( {{\text{Ca}},{\text{ Mg}}} \right)^{{{2} + }}$$

Metal ions have to exchange exchangeable cations (Na^+^, K^+^, Ca^2+^, and Mg^2+^) via passing through the pore spaces and openings of zeolite during the ion exchange process. As a consequence, the degree to which Al is substituted for Si in the framework determines a zeolite’s cation exchange capability. Therefore, the more cations demanded to keep the zeolites’ electrical neutrality, the more Al ions must be substituted for Si ions. These cations that balance charges have the ability to exchange with other cations present in the solution^14^. The main objective of this work was to identify the ability of NZ (clinoptilolite) and SZ (Faujasite) to remove studied metallic ions (i.e. (Cr, Ni, V, and Zn) from heavy crude oil (BD) that through studying the parameters that affect demetallization process rate i.e., contact time, zeolite as adsorbent load, crude oil concentration, as well as determine the maximum removal efficiency of zeolites (NZ and SZ) to remove the studied metal ions through reaching to equilibrium conditions. Also comparing between zeolites (NZ and SZ) used in this work to recognize which type more effective and efficient to demetallization process of BD.

## Methodology

**Fig. 1:**
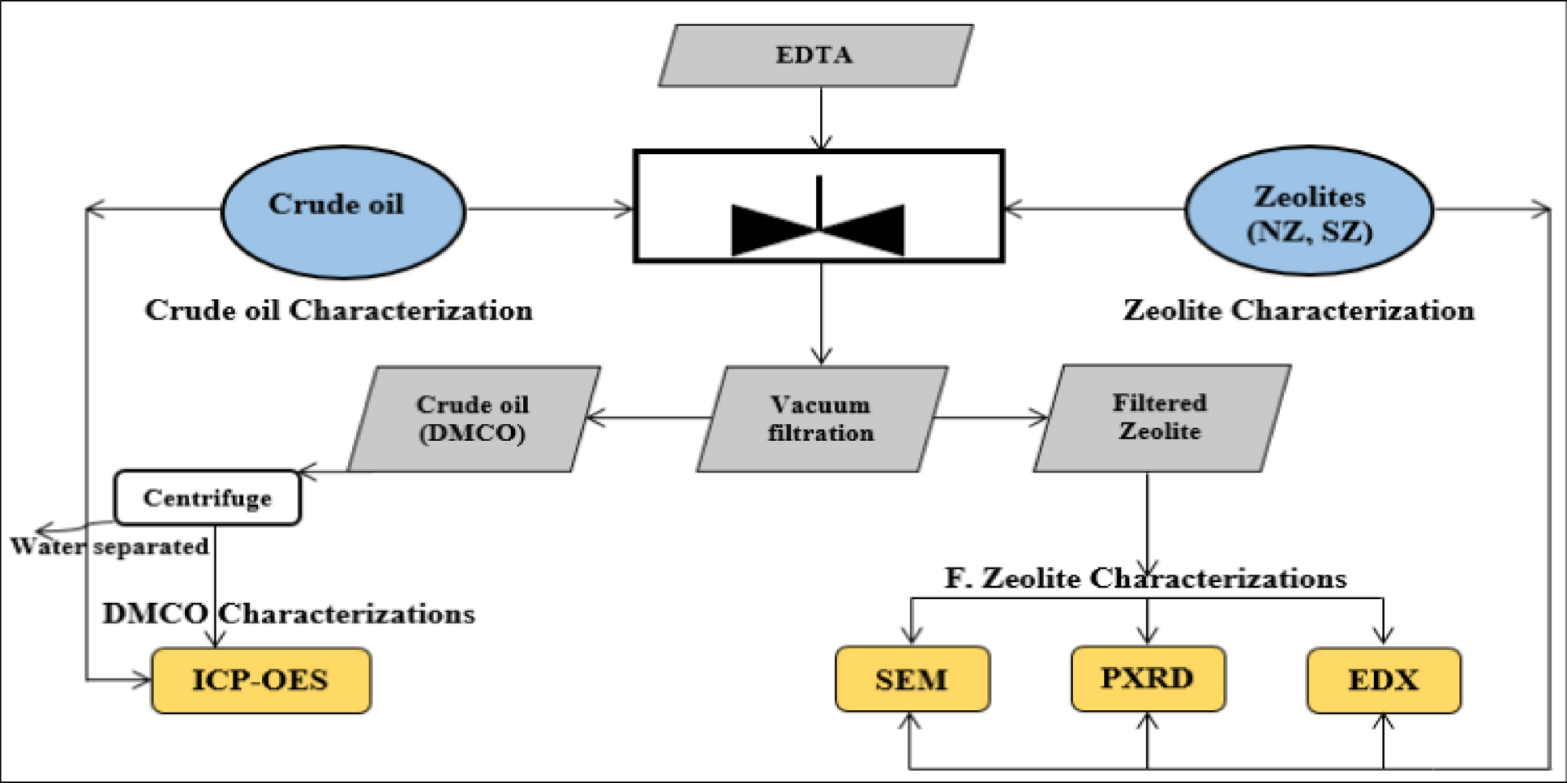
Flow chart represents demetallization process of crude oil (BD).

This work has demonstrated the demetallization of crude oil (BD) in several practical manners. The characterization of the crude oil (BD) and the zeolites (NZ and SZ) that were utilized were the first practical steps to be completed. The experimental demetallization technique proceeded on to the examination of the crude oil samples and the zeolites that were left over after the extraction process. Figure 1 shows the flowchart of these useful procedures.

### Materials

Clinoptilolite [Natural Zeolite (NZ)] (Ca, K_2_, Na_2_, Mg)_4_Al_8_Si_40_O_96_.24H_2_O was obtained from Taiz area, Yemen, Faujasite [Synthetic Zeolite (SZ)] (SiO_2_:Al_2_O_3_) zeolite Y, sodium was purchased from Alfa Aesar, Crude oil [Belayim Desert crude oil (BD)] was received from Belayim oil field, Egypt, Ethylenediaminetetraacetic acid disodium salt dehydrate (EDTA) was purchased from SIGMA-ALDRICH, UK, HCl & NaOH flakes to adjust solution pH range.

### Zeolites preparation for characterization

Natural Zeolite (NZ) was crushed by a jaw crusher for 10 min. then it sieved using a mechanical sieve shaker for 15 min. to obtain NZ grain size ≈ 150 µm. It washed by a continuous stream of Demi-water for 5 min. to remove all fine cracked particles found inside pore spaces and dried at 110 °C for 8 h. collected again to be ready for use. While Synthetic zeolite (SZ) was purchased in powder, grain size (≈ 75 µm.) didn’t need for preparation before use. A number of techniques have been applied in this study to explain the characteristics of raw zeolites both before and after the demetallization process. These methods include: Zeolites’ mineralogical composition was ascertained by Powder X-Ray Diffraction (PXRD), (type X-Ray Tube Cu 1.54060 A., 40.0 keV, current 30.0 mA) D2 PHASER, Benchtop X-ray Powder Diffraction, Bruker, Germany. Utilizing energy-dispersive X-ray spectroscopy (EDX) techniques, and the chemical structure of zeolites was studied. Zeolites’ surface morphology was investigated using a scanning electron microscope JEOL JSM-IT200 Scanning Electron Microscope (SEM–EDX), Japan. Both before and after the demetallization process. In order to match the collected data with the purchased zeolite data, the elemental composition of the zeolites has been identified using Induced Coupled Plasma Optical Emission Spectrophotometry (ICP-OES) (Varian Inc.-Vista-PRO). At 700 °C, zeolites are ash-treated and then combined with lithium borate. After dissolving the melt in diluted hydrochloric acid (HCl), the resulting solution is subjected to simultaneous or sequential multi-elemental element analysis by ICP-OES^15^.

### Heavy metals measurements

ICP-OES (Varian Inc.-Vista-PRO) was used to determine the metal content of Belayim Desert crude oil (BD) both before and after the demetallization process. After treating the crude oil samples to lessen the metals volatility, they are coked and ashed. After applying aqua-regia (1 ml of concentrated HNO^3^ and 3 ml of concentrated HCl), the residue evaporates and is then dissolved in acids. As an internal standard, scandium is added. ICP-OES is used to determine the elemental concentrations in the final solutions that are produced^16^.

### Demetallization process

**Fig. 2:**
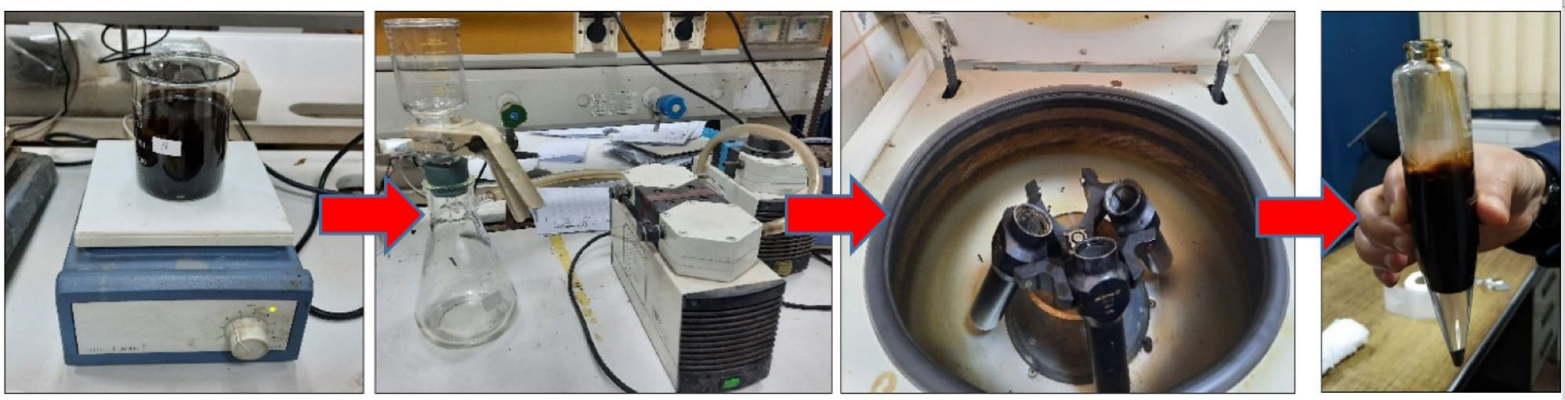
Demetallization procedure of crude oil.

Demetallization procedure of crude oil for adsorption of metal ions that illustrated in Fig. 2 includes; mixing different crude oil quantities 50, 100, 150 and 200 ml. with NZ at different concentrations 0.5, 1, 2, 3 and 4 g in a glass beaker with a 100 ml. of EDTA solution. A magnetic stirrer set to 300 rpm was used for thoroughly combining the mixture over the course of 3, 6, 12, 18, and 26 h at room temperature (about 25 °C). All prior processes were repeated, but with SZ. Using vacuum filtration machine each type of zeolites (NZ and SZ) has been separated from the mixture. The liquid mixture was centrifuged in a high-speed centrifuge (SIGMA Co.) set at 1600 rpm for 30 min. This allowed the de-metalized crude oil (DMCO) layer to be separated from the aqueous layer of EDTA. The DMCO layer was set aside for the analysis of metallic ions using the ICP-OES technique. Depending on previous studies based on study the effect of pH on the absorption process; the published data^17,18^ the best pH range of adsorption of heavy metals onto zeolite has been varied between 3 and 6. In the present study, the selected pH was ~ 4.5, adjusted using NaOH and HCl.

#### Factors influencing on demetallation process

Demetallation process of heavy crude oil is very complicated in relation to several factors that effect on the general reaction rate. Among these include contact time (stirring time), Zeolites load (concentrations of adsorber NZ and SZ in gram) and initial metal ions concentration (crude oil quantity in ml.).

##### Effect of contact time

The contact time was investigated separately for all treated crude oil samples using NZ and SZ at different time intervals 3, 6, 12, 18 and 26 h. at a fixed adsorbent concentration of NZ and SZ (0.5 g.). so, the total solution volume used was 150 ml. comprising of 1:2 ratios of crude oil/EDTA solution (aqueous solution); 50 ml. crude oil: 100 ml. aqueous solution. It was mixed using a magnetic stirrer set at a fixed speed 300 rpm.

##### Effect of load dosage (NZ and SZ adsorbent concentration)

The effect of adsorbent was carried out at different concentrations of 0.5, 1, 2, 3 and 4 g. of each adsorbent (NZ and SZ respectively) using the same 1:2 ratios of crude oil/EDTA solution (aqueous solution); 50 ml. crude oil: 100 ml. aqueous solution respectively. It was mixed using a magnetic stirrer set at a fixed speed 300 rpm.

##### Effect of initial metal ions concentration (BD crude oil quantity)

At a set concentration (0.5 g.) of the adsorbent (NZ and SZ), the influence of the adsorbate concentration (amount of BD crude oil) on the sorption of metal ions was investigated. The ratios of crude oil/EDTA solution (aqueous solution) were varied to 1:2, 1:1, 3:2, and 2:1 ml, respectively, at a fixed contact time.

### Equilibrium adsorption

The ratio of metal ions (%) absorbed by the adsorbent relative to the total amount of metal ions in the aqueous solution at the start of the process. Equations (3) or (4) were used to calculate the efficiency as a percentage of metal ions removed:

3 $${\text{Efficiency}}\;{\text{of}}\;{\text{metal}}\;{\text{removal}}\;\left( \% \right) = \left( {{\text{C}}_{{\text{i}}} {-}{\text{C}}_{{\text{t}}} } \right)/{\text{C}}_{{\text{i}}} \times {1}00$$

or (under conditions of equilibrium);

4 $${\text{Efficiency}}\;{\text{of}}\;{\text{metal}}\;{\text{removal}}\;\left( \% \right) = \left( {{\text{C}}_{{\text{i}}} {-}{\text{C}}_{{\text{e}}} } \right)/{\text{C}}_{{\text{e}}} \times {1}00$$

where C_i_ (mg. L^-1^) and C_t_ (mg. L^-1^) indicate to the initial and residual metal concentration acted in the solution at specific time (t), respectively. In a number of texts^19^, C_i_ is indicated as C_0_. Under condition of equilibrium Ct = Ce. The likelihood and selectivity of an adsorbent (zeolites) in extracting a particular metal ion from a solution within a specified time frame are elucidated by adsorption efficiency^20^.

## Results and discussion

### Kinetic study of adsorption

**Fig. 3:**
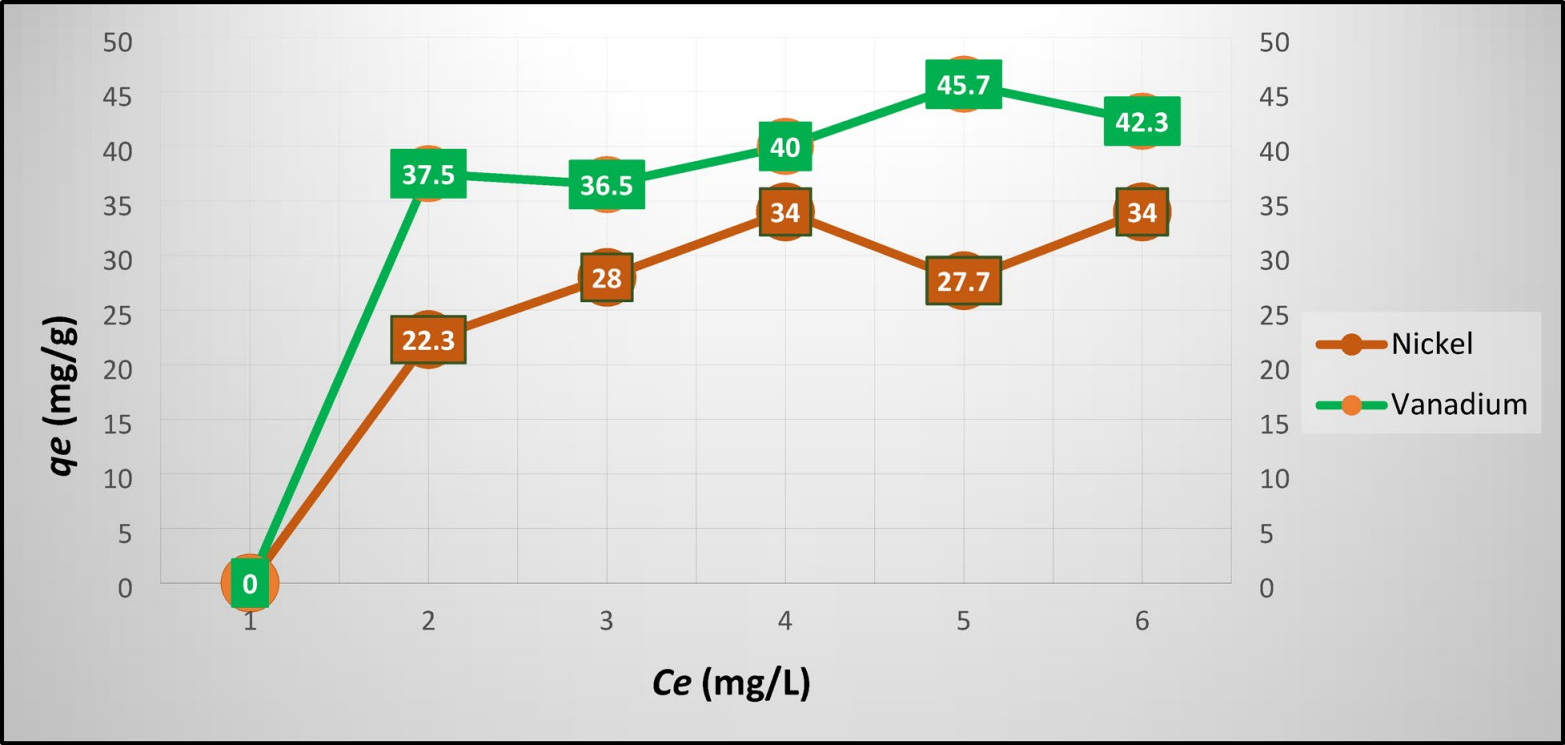
Experimental results of Ni/V adsorption at equilibrium on NZ.

**Fig. 4:**
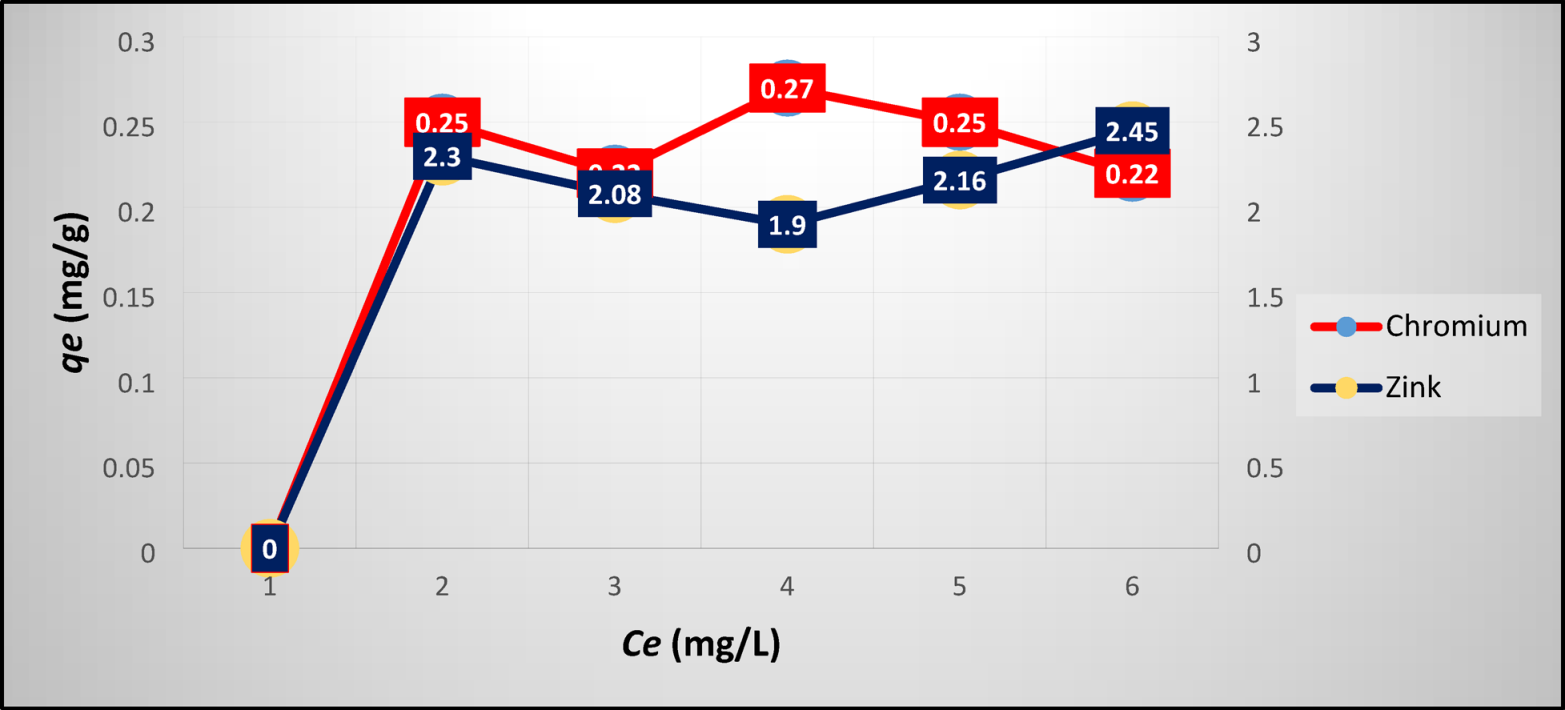
Experimental results of Cr/Zn adsorption at equilibrium on NZ.

The number of metals adsorbed at equilibrium, (mg/g) was estimated using Eq. (5).

5 $$qe=\frac{\left(C0-Ce\right) V}{W}$$

where C_0_ and C_e_: concentrations of metal at initial and at equilibrium (mg/L), respectively, V: volume of the heavy oil used, and W: mass of zeolite (NZ, SZ) used (g). Figures 3 and 4 plot the experimental results of Ni/V and Cr/Zn adsorption at equilibrium on NZ, respectively. Figure 3 depicts that the maximum uptake of Ni/V metals were ≈34/45 mg g^-1^, this may be attributed to the capability of the NZ framework which having a high percentage of pores.

**Fig. 5:**
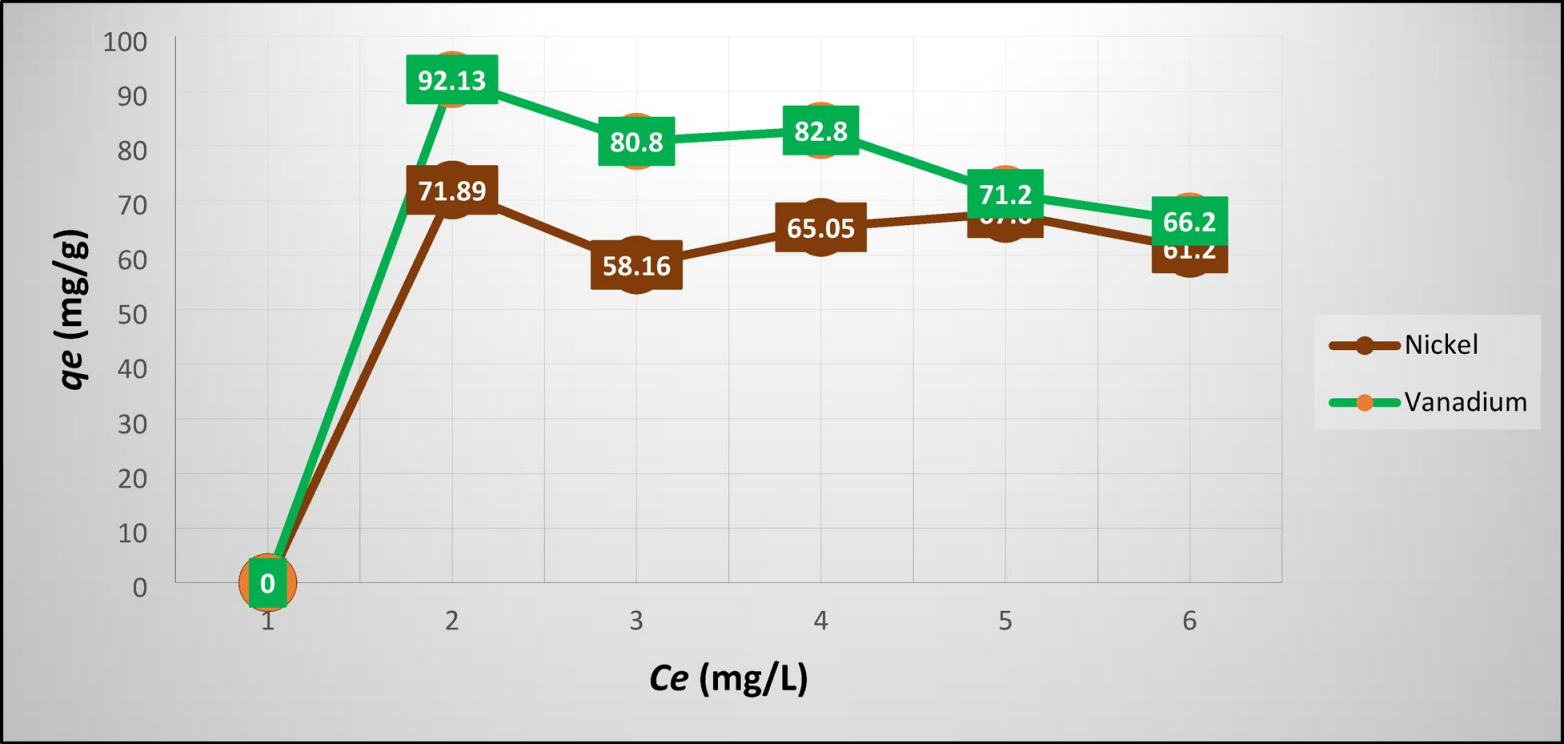
Experimental results of Ni/V adsorption at equilibrium on SZ.

**Fig. 6:**
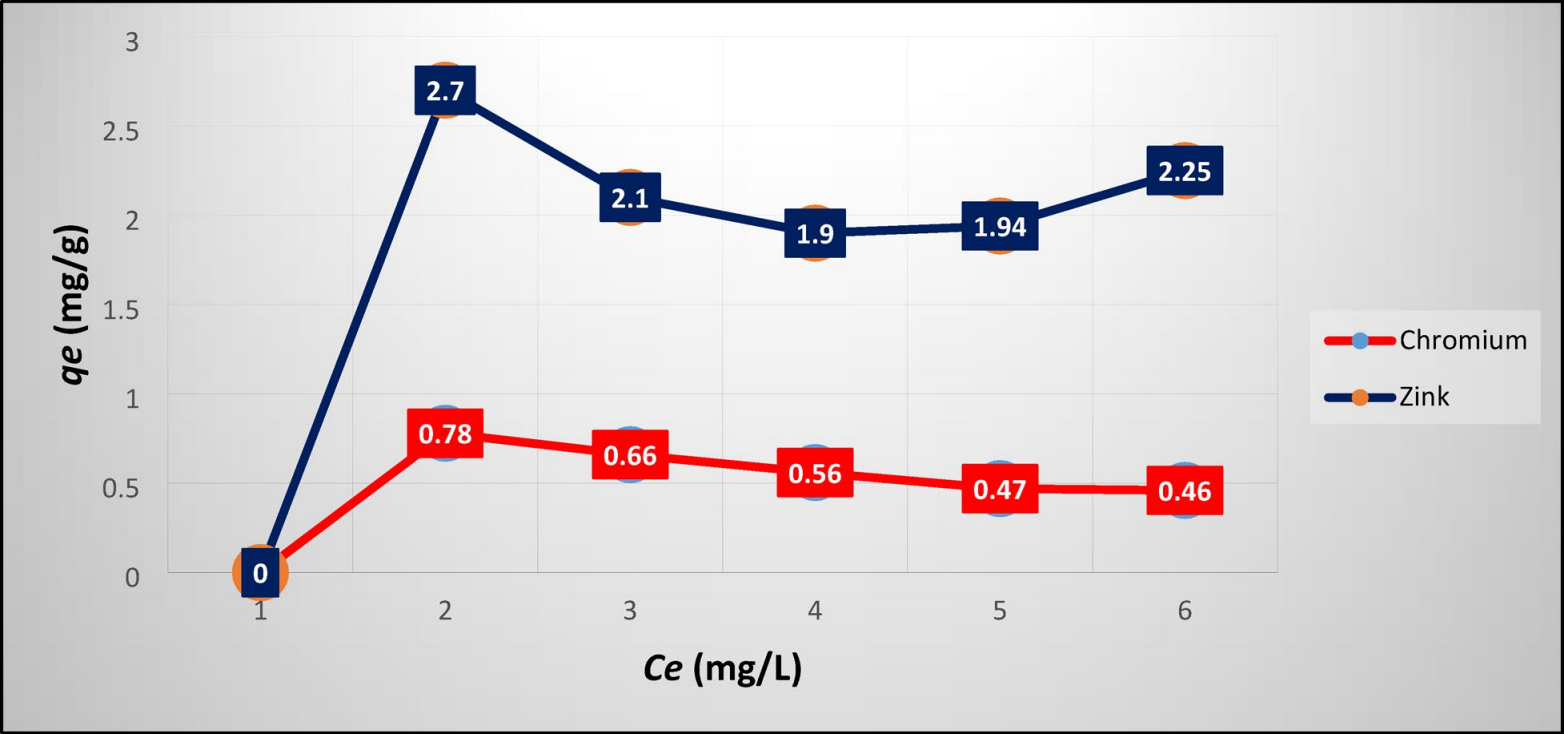
Experimental results of Cr/Zn adsorption at equilibrium on SZ.

Figure 5 and 6 plot the experimental results of Ni/V and Cr/Zn adsorption at equilibrium on SZ, respectively. Figure 5 depicts that the maximum uptake of Ni/V metals were ≈72/92 mg g^-1^ this may be attributed to the capability of the SZ framework which having a high percentage of pores.

The Freundlich and Langmuir equations are the most widely used models for isotherm^17^, the correlation of Freundlich and Langmuir isotherms are represented by Eqs. (6) and (7), respectively.

6 $$qe = KF Ce^{1/n}$$

7 $$qe=\frac{qm . KL . Ce}{(1+KL . Ce)}$$

where qm: maximum quantity of metals adsorbed per unit mass of zeolite (mg. g^-1^), qe: concentricity of metals at equilibrium (mg. L^-1^). KL is the Langmuir constant (L mg^-1^) and KF is Freundlich constant (mg g^-1^) (L mg^-1^)^-n^. To predict which one of these two models will well represent the experimental data of metals removal, a linearization technique would be applied on Eqs. (6) and (7), respectively. Equations (8) and (9) represent the linearized form for Eq. (6) and (7) respectively.

8 $$\frac{1}{qe}=\frac{1}{qo}+\frac{1}{bqmCe}$$

9 $$\text{ln}{q}_{e}=\mathit{ln}{k}_{F}+\frac{\mathit{ln}{c}_{e}}{n}$$

**Fig. 7:**
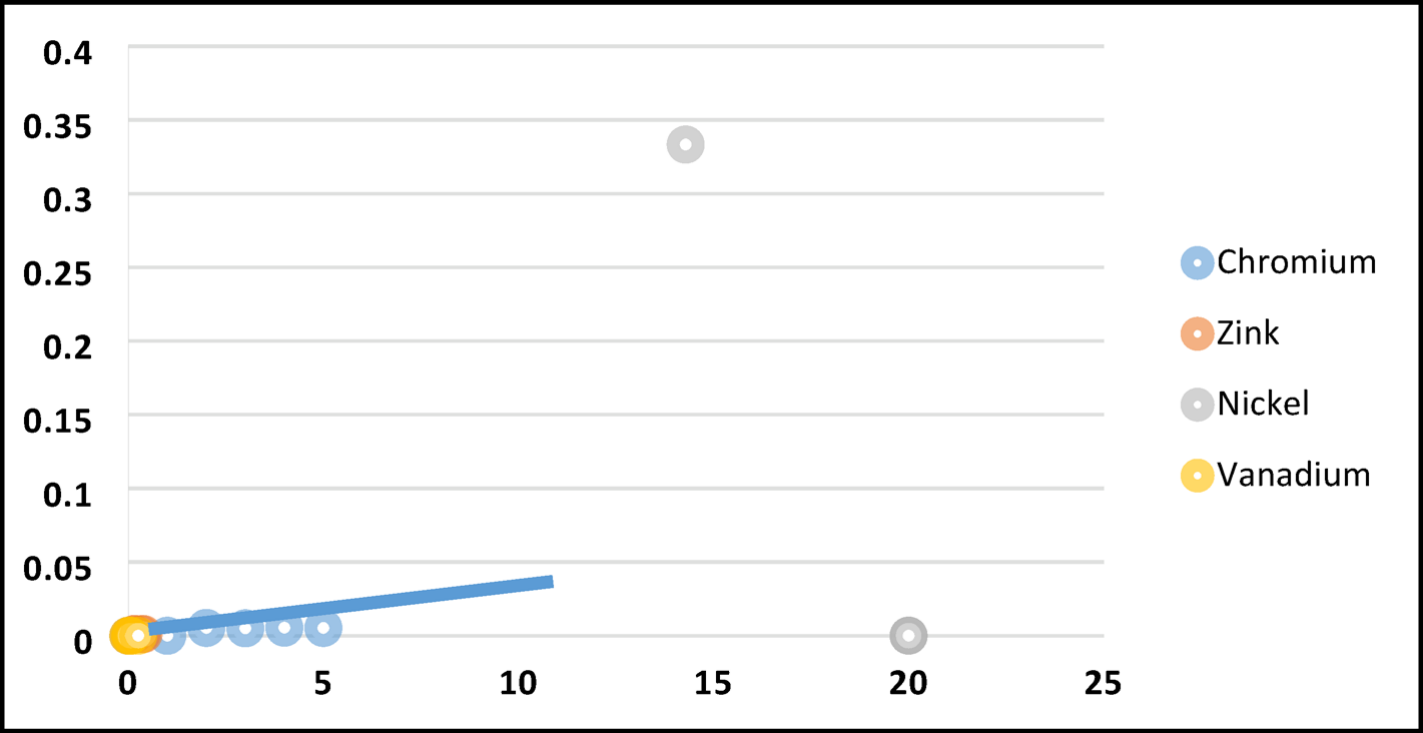
Linearization plots of Langmuir isotherm model for Ni, V, Cr and Zn adsorption on NZ.

**Fig. 8:**
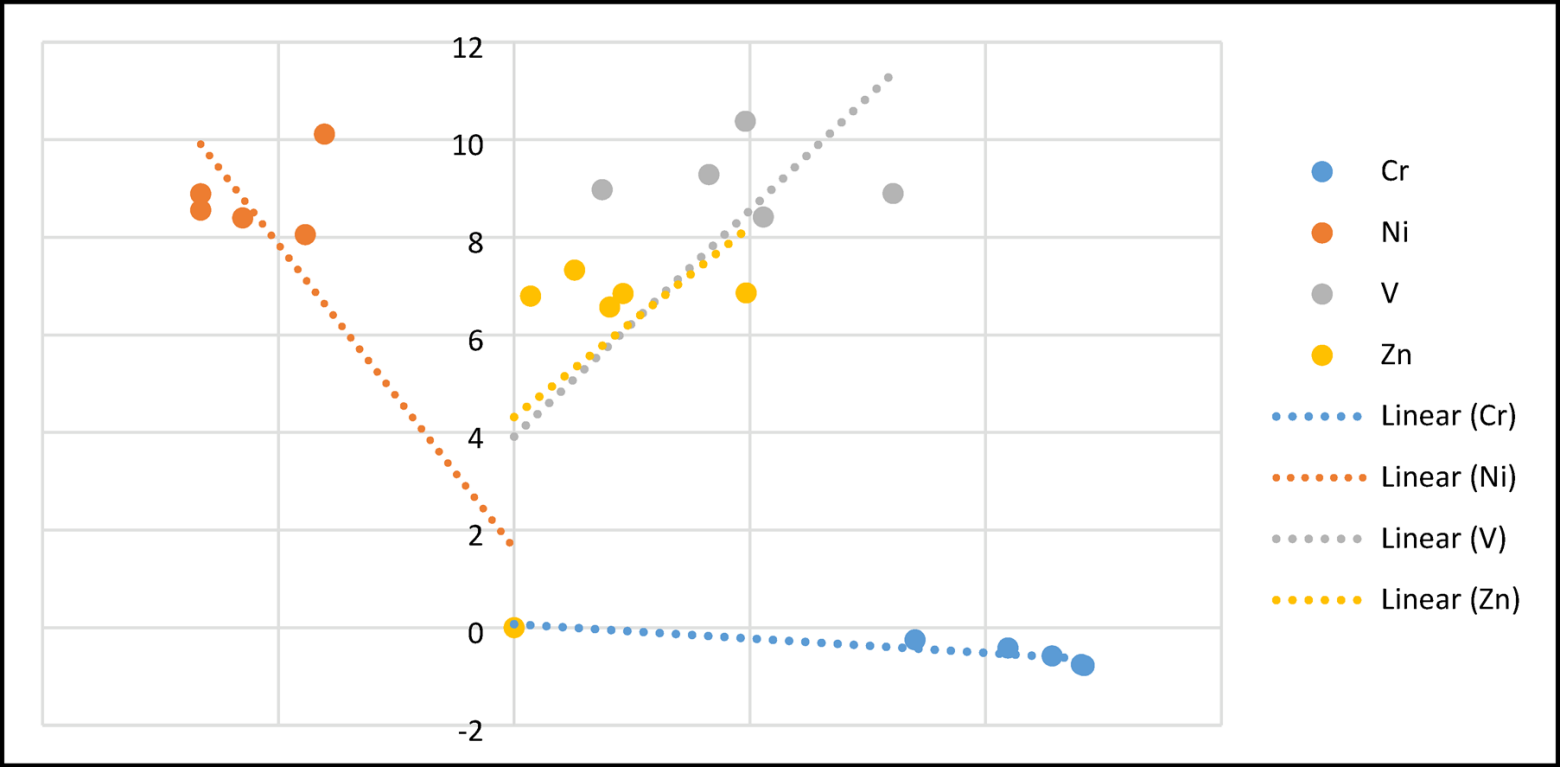
Linearization plots of Freundlich isotherm model for Ni, V, Cr and Zn adsorption on SZ.

Figure 7 represents a plot of $\frac{1}{qe}$ vs $\frac{1}{Ce}$ with a correlation coefficient (R^2^) = 0.79812. Meanwhile Fig. 8 represents a plot of $\mathit{ln}{q}_{e}$ vs $\mathit{ln}{c}_{e}$ with a correlation coefficient (R^2^) = 0.71542. These results assure the viability of Langmuir isotherm in present study. This outcome depicts that the maximum uptake of Ni/V metals were ≈72/92 mg g^-1^, this may be attributed to the capability of the usable of NZ and SZ structural frame with high percentage of pore volume.

### Characterization of BD crude oil

In addition to the hydrocarbon fingerprinting of the crude oil type (BD) employed in this work, general characterization investigations were conducted on the crude oil (BD) sample to ascertain the physical–chemical characteristics using ASTM standard test techniques, as indicated in Table 1.

**Table 1:** Physical–chemical characteristics of Belayim crude oil (BD).

Test	Method	Units	Crude oil (BD)
Density @ 15 °C	ASTM D 5002^21^	kg/m^3^	0.9249
API gravity	Calculated	Unit less	21.49
Kin. viscosity @ 40 °C	ASTM D 445^22^	cSt	25.296
Total sulfur	ASTM D 4294^23^	% wt	3.15
Salt content	ASTM D 3230^24^	PTB	13
Water	ASTM D 4928^25^	% vol	0.15
Carbon residue	ASTM D 4530^26^	% wt	9.20

#### Crude oil ICP-OES analysis

Table 2 illustrates ICP-OES analysis of crude oil (BD) before demetallization process, that this analysis revealed the most abundant metal ions in BD are Vanadium and Nickel ions then Zinc concentration ion and Chromium ion the least one, so this study focused on these target metal ions for demetallization process of BD. The fact that most soils associated with areas where crude oils are found in Egypt (South Sinai) have notable concentrations of metal ores such as zinc ores, vanadium, and nickel, which are commonly associated with such ores, could account for the relatively high concentration of nickel and zinc in the Belayim Desert (BD) crude oil form compared to the other metals investigated.

**Table 2:** Metals in Belayim desert crude oil (BD) using ICP-OES analysis.

Test	Method	Units	Crude oil (BD)
Cr	UOP 389^16^	mg/kg	0.88
Ni			82.4
V			107.2
Zn			5.08

### Characterization of zeolites

#### Zeolites ICP-OES analysis

Table 3 presents the elemental analysis of NZ and SZ used in this work by ICP-OES, the table presents elemental composition in wt. % of zeolites (NZ and SZ) in metal oxides form. In NZ type, it consists mainly of Silica (≈ 63%), Aluminum (≈ 16%), and traces from Calcium, Potassium, Iron, and other elements. On the other hand, in SZ type, consists mainly of Silica (≈ 56%), Aluminum (≈ 24%), Sodium (≈ 19%), and other elements.

**Table 3:** ICP-OES elemental analysis of NZ and SZ.

Oxides	NZ (wt. %)	SZ (wt. %)
SiO_2_	63.11033	55.7173
Al_2_O_3_	16.27557	24.39514
CaO	6.80173	0.155607
K_2_O	5.295632	0.281096
Fe_2_O_3_	5.247049	0.105411
Na_2_O	1.943351	18.82341
MgO	1.068843	0.050196
ZnO	0.111743	0.130509
CuO	0.063159	0.065254
Cr_2_O_3_	0.048584	0.200783
NiO	0.024292	0.060235
PbO	0.009717	0.015059
Total	100%	100%

#### X-ray diffraction analysis

**Fig. 9:**
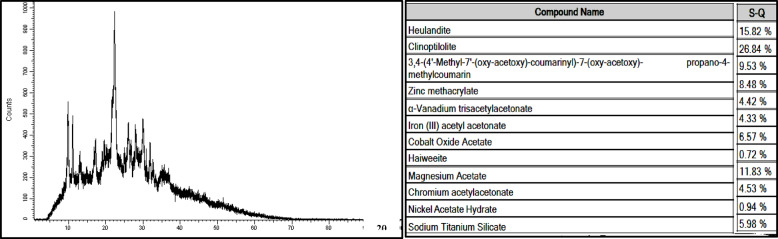
XRD analysis of NZ.

**Fig. 10:**
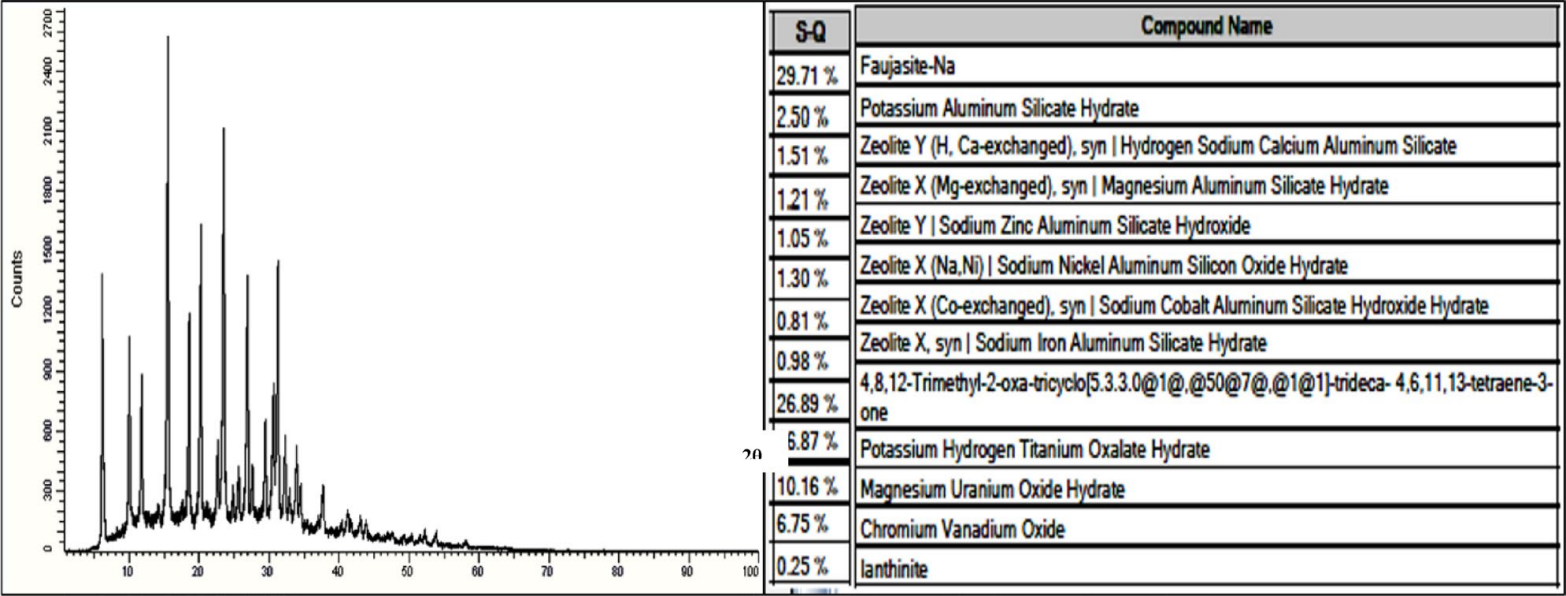
XRD analysis of SZ.

Figures 9 and 10 show XRD analysis for NZ and SZ respectively with name compounds table behind, according to Combination of mannered XRD Powder Patterns for Zeolites^27^, the results confirmed that there is a great match between typical Clinoptilolite and Faujasite, respectively and International Center for Diffraction Data (ICDD) 47–1870 data library, that discover the targeted zeolites, both synthetic and natural, were presented as the same targeted zeolites by the formation of the targeted zeolite framework structures. The zeolites’ PXRD patterns indicate that all of the materials crystallize as a single crystalline phase with a high degree of crystallinity, as evidenced by the close match between the ICDD database and the observed patterns. The close match between the ICDD database and the observed patterns in the PXRD patterns of the zeolites suggests that all of the materials form as a single crystalline phase with a high degree of crystallinity. Compounds that make up the key mineralogical composition of NZ and SZ, as determined by PXRD review, are mentioned in figures.

#### SEM–EDX analysis

**Fig. 11:**
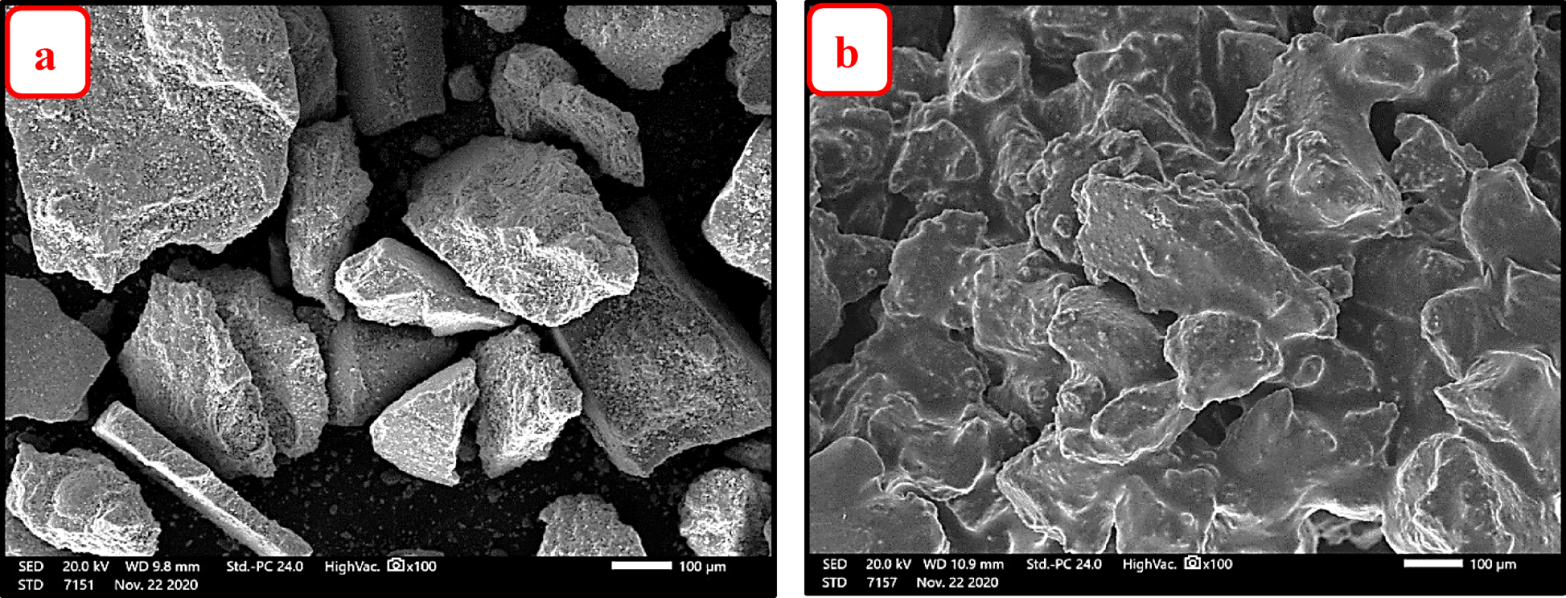
SEM image of NZ (**a**) before demetallation (**b**) after demetallation process.

**Fig. 12:**
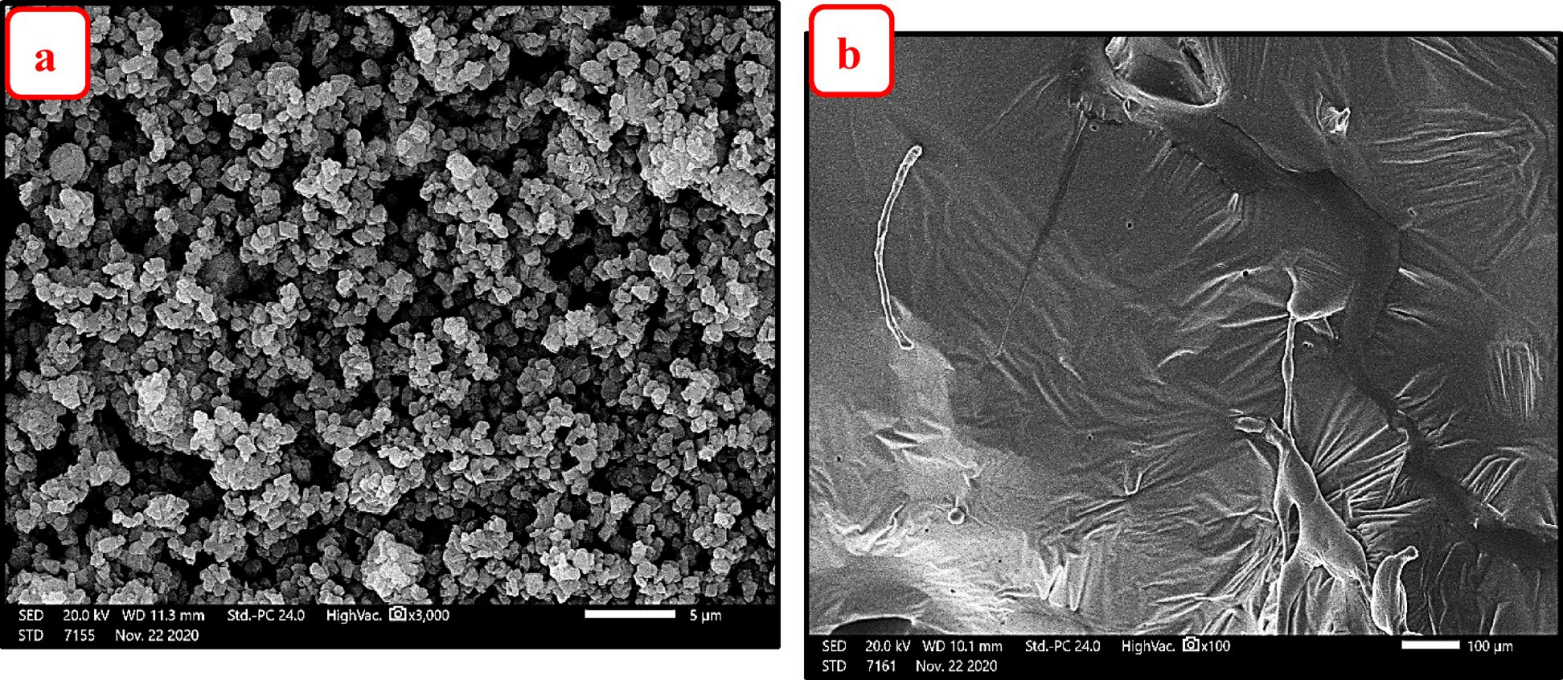
SEM image of SZ (**a**) before demetallation (**b**) after demetallation process.

Figure 11a,b illustrate the surface morphologies (SEM images) of NZ before and after demetallation process respectively, while Fig. 12a,b show the surface morphologies (SEM image) of SZ before and after demetallation process respectively.

EDX analysis of the zeolites (NZ and SZ) were carried out, before and after demetallation process to determinate the wt. % of elemental composition. Also, to determine the ratio of Si/Al in the zeolites (NZ and SZ) before and after demetallation process. The zeolites NZ and SZ’s Si/Al ratios (before process) were; 4.74 and 2.73% respectively, the zeolites NZ and SZ’s Si/Al ratios NZ and SZ (after process) were; 5.02 and 1.68% respectively. The percentage mass of elements as determined by the SEM–EDX and the Si/Al ratios in zeolites (NZ and SZ), respectively, are shown in Tables 4 and 5. An increase in this ratio indicates a greater adsorption capacity of the adsorbents^14^.

**Table 4:** Percentage mass of elements before and after demetallation process in NZ.

% wt	C	O	Na	Al	Si	Si/Al	S	Ni	Zn
	Before/after	Before/after	Before/after	Before/after	Before/after	Before/after	Before/after	Before/after	Before/after
NZ	3.77/61.09	54.73/22.11	0.38/0.77	5.95/2.06	28.20/10.34	**4.74/5.02**	0.0/1.08	0.0/0.14	0.0/0.16

Significant values are in [bold].

**Table 5:** Percentage mass of elements before and after demetallation process in SZ.

% wt	C	O	Na	Al	Si	Si**/**Al
	Before/after	Before/after	Before/after	Before/after	Before/after	Before/after
SZ	4.64/55.72	51.96/28.14	9.04/2.04	9.22/5.26	25.14/8.84	**2.73/1.68**

Significant values are in [bold].

**Fig. 13:**
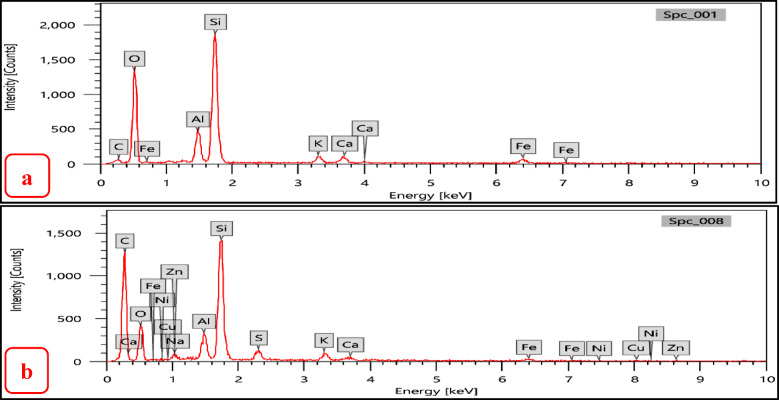
EDX analysis (**a**) NZ before demetallation, (**b**) after demetallation process.

**Fig. 14:**
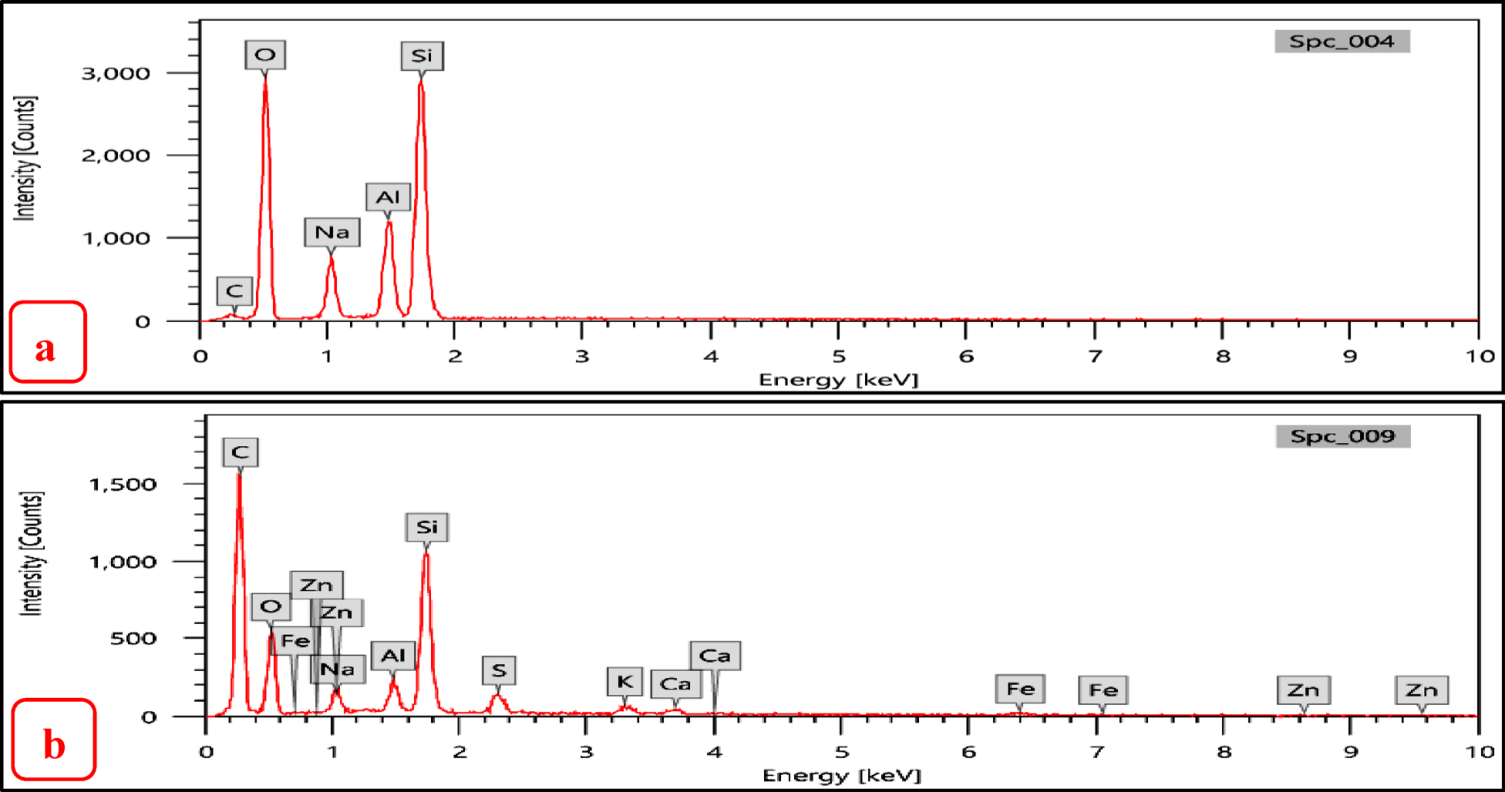
EDX analysis (**a**) SZ before demetallation, (**b**) after demetallation process.

As can be seen in Figs. 13a,b and 14a,b show EDX graphs of NZ and SZ before and after demetallization process, respectively. New elements occur in EDX analysis after demetallization process like (Cu, Ni, S, and Zn) these elements should be extracted from crude oil (BD) which give great evidence for the efficiency of zeolite to extract these metals from crude oil, note dramatically increasing in Carbon element (C) concentration from figure “a” to “b”, due to completely homogeneity (mixing) between zeolite and crude oil (H/C) represent source of carbon in EDX analysis after demetallization process (residual crude oil on the surface of zeolite). It was observed that some of Al and Si inside the zeolites were replaced and moreover, around 63.33% of Si ions (in NZ) and 64.8% of Si ions (in SZ) which forms the main structure of zeolites were replaced by ions of Cu, Ni, S, and Zn on the zeolite surface, while about 65.3% of Al ions (in NZ) and about 43% of Al ions (in SZ) were replaced.

According to Gomes et al.^28^, ion radius has a significant impact on ionic exchange. Shannon^29^ released updated crystallographic information regarding ionic radii. According to his list (see also www.abulafia.mt.ic.ac.uk/shannon), the ionic radii of Na^+^, Zn^2+^, Ni^2+^, Al^3+^, Cr^2+^, Si^4+^, and V^4+^ are 102, 60, 49, 48, 41, 40, and 35.5 Pico-meters, respectively. The significant influence of Al and Si ions exchanged with the framework and the collapse of the zeolite structure observed after the demetallation process can be explained by the extracted ions’ ability to exchange any ions of equal or bigger size, as indicated by these numbers.

### Results of demetallization process

#### Effect of contact time

**Fig. 15:**
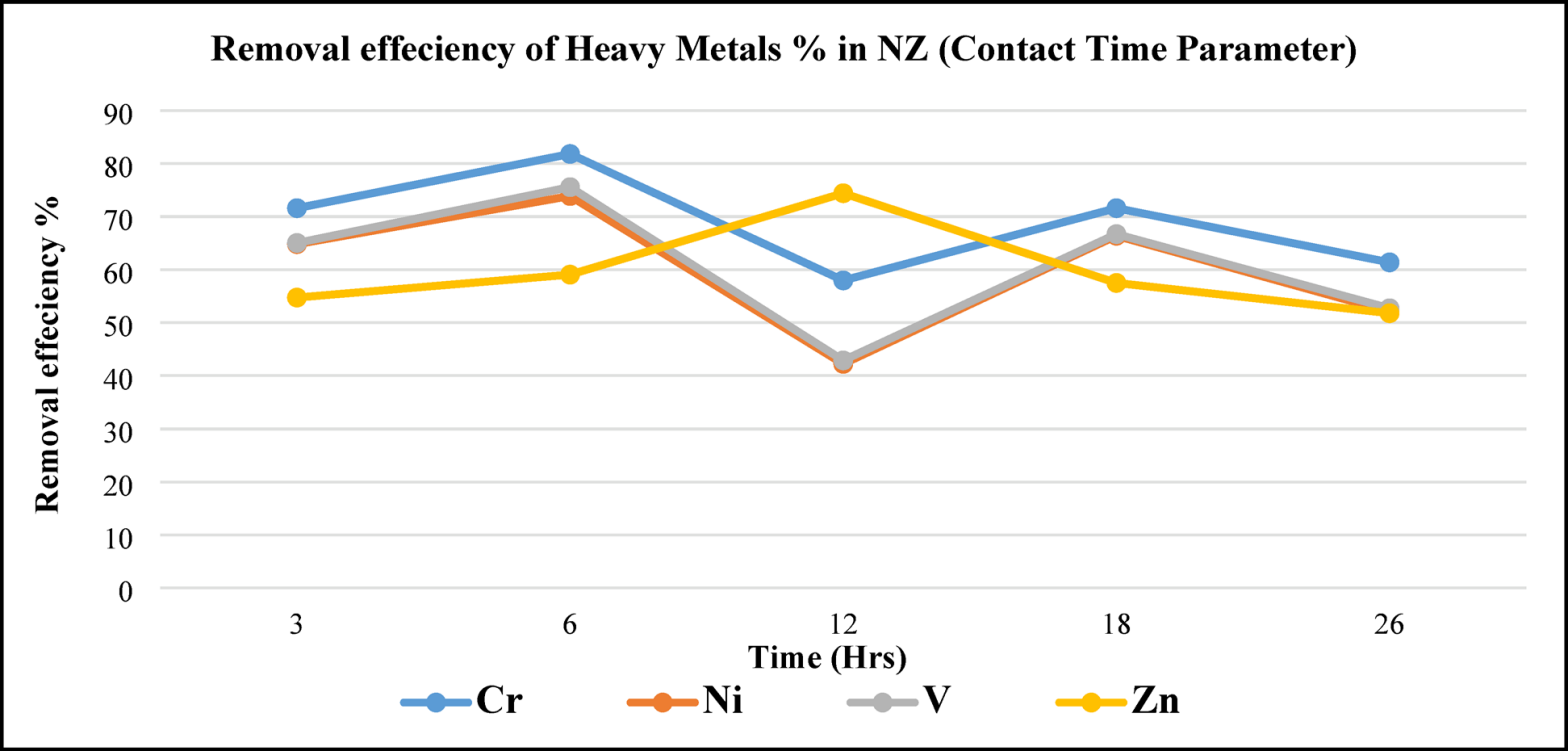
Metals removal efficiency from BD as a function of contact time using NZ.

Figure 15, illustrates (Cr, Ni, V, and Zn) metals removal efficiency of metals using NZ as a function of operating time due to different time intervals. A remarkable strong matching between vanadium and nickel removal efficiency with time contact, and the same efficiency pattern for Cr where increasing efficiency from 3 to 6 h. then decreased at 12 h. and continually efficiency increasing at 18 to 26 h. Maximum removing efficiency for V, Ni, and Cr recorded at 6 h. contact time, while minimum efficiency recorded at 12 h. contact time. On the other hand, Zn removing efficiency slightly increasing 3 to 6 h. to records maximum efficiency at 12 h. and efficiency returns decreasing slightly again. Consider, the optimum metal removal efficiency obtained at 6 h. contact time, so it is used as a fixed parameter (6 h.) to complete experiments in the next experimental parameter (NZ load).

Thus far, this contact time has lined up with Luma and Mohammad’s (2019) findings^30^. According to experiments, the removal efficiencies of vanadium for zeolites loading at 1 g/100 mL oil and in around 5 h^17^ were 60, 45, and 33% for loadings of 75, 85, and 95 ppm, respectively. After 10, 20, 40, and 50 h, removal efficiency for 75 ppm of V packing was 68, 75, 78, and 78%.

**Fig. 16:**
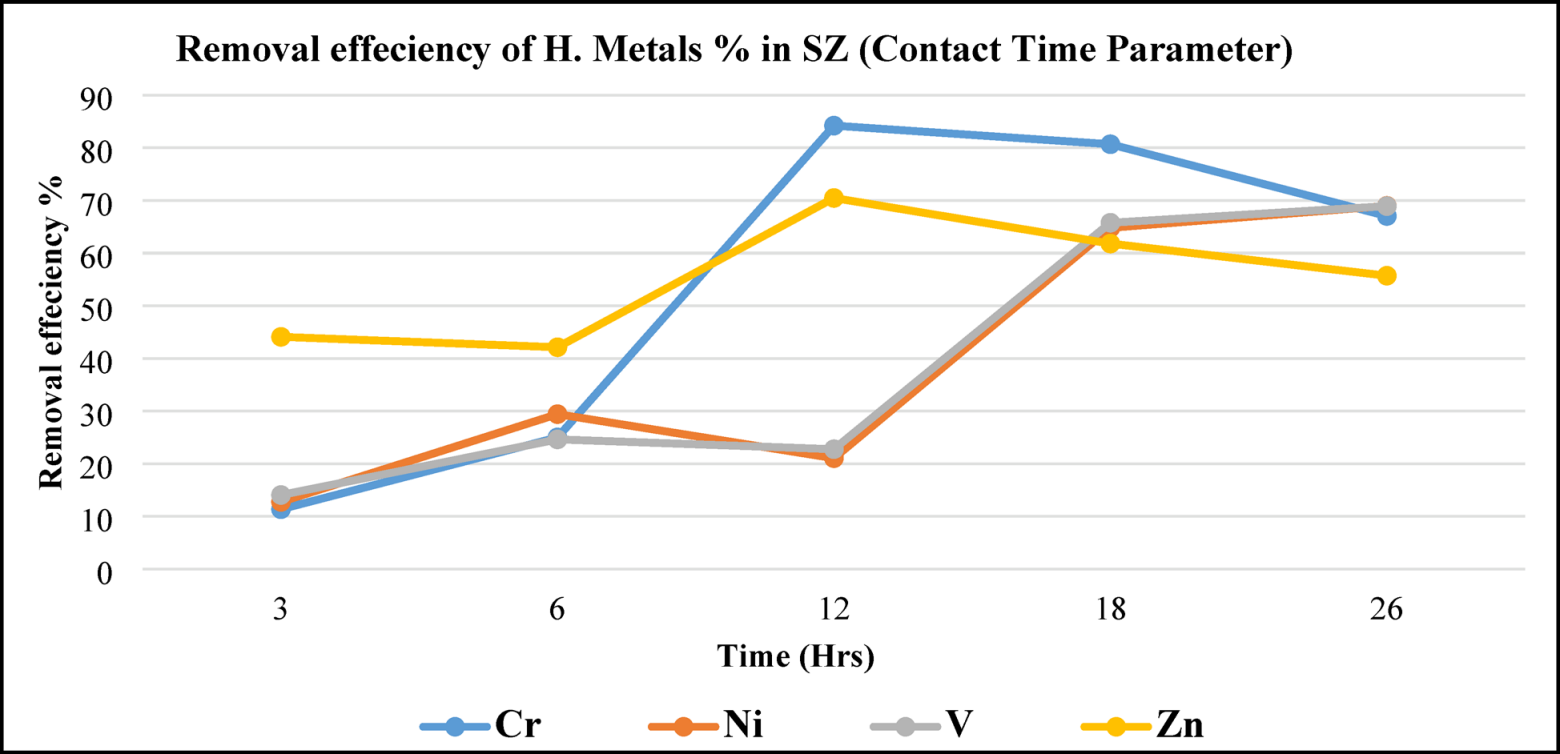
Metals removal efficiency from BD as a function of contact time using SZ.

Figure 16, illustrates (Cr, Ni, V, and Zn) metals removal efficiency of metals using SZ as a function of operating time due to different time intervals. Maximum removing efficiency for Vanadium and Ni recorded at 26 h. contact time, while minimum efficiency recorded at 12 h. contact time. On the other hand, Zn removing efficiency records maximum at 12 h. and efficiency returns decreasing slightly again 18 to 26 h. Consider, the optimum metal removal efficiency obtained at 18 h. contact time, so it is used as a fixed parameter (18 h.) to experiment with the next experimental parameter (SZ load). By using SZ, the time contact parameter should be increased to obtain high removal efficiency. So, it is clear highly removal efficiency by using SZ occurred in 12 and 26 h. contact time.

##### Effect of load dosage (NZ and SZ adsorbent concentration)

**Fig. 17:**
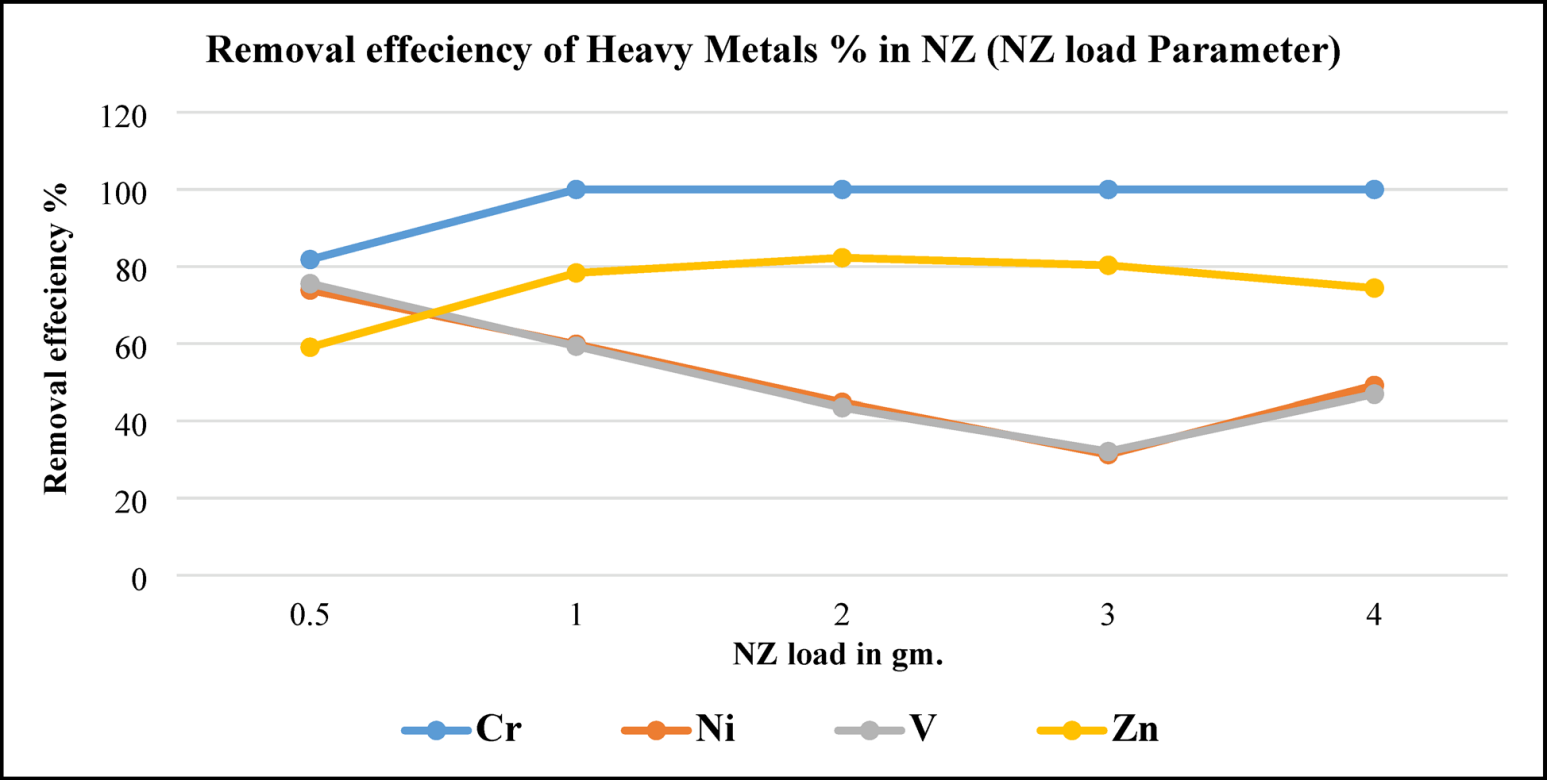
Metals removal efficiency from BD at NZ load parameter.

Figure 17, illustrates metals removal efficiency by using different NZ masses. As can be seen in this figure, Cr removing efficiency increased from 82 to 100% by increasing NZ load. On the other side removing efficiency of Ni and V pattern completely matched together, by increasing NZ load removing efficiency decreased till to using 3 g. of NZ then increased slightly to 49% at 4 g. of NZ. Results were against the results were obtained by Luma and Mohammad^30^ that reported, zeolite loading improves the performance of removal. It’s worth noting that at higher NZ (4 g.) loadings, the amount of vanadium and nickel removed was kept constant, then increased. That may be due to a balance being formed between the adsorbed vanadium and nickel ions on NZ and the ions that remained in the treated crude oil.

**Fig. 18:**
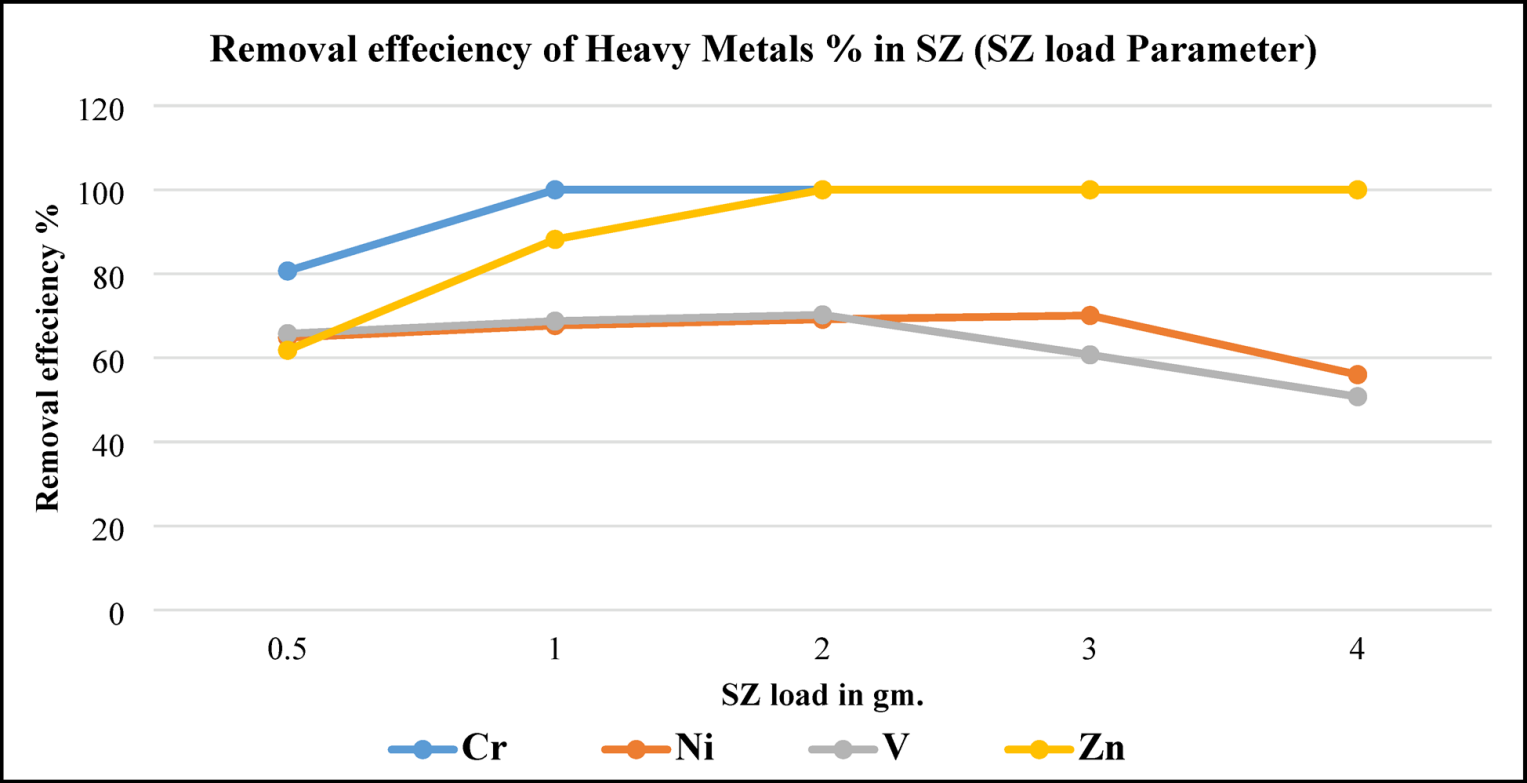
Metals removal efficiency from BD at SZ load parameter.

Figure 18, illustrates metals removal efficiency by using different SZ masses. As can be seen in this figure, Cr and Zn removing efficiencies were increased from 82 to 100% and 62 to 100% respectively by increasing SZ load. This implied that equilibrium was reached quickly at 1 g. of SZ. These results agree with results were obtained before by^31^. On the other side removing efficiencies of Ni and V were increased gradually by increasing SZ load till to using 3 g. of SZ then decreased slightly. Results comply with the results were obtained by Luma and Mohammad^30^, reported that, zeolite loading has a positive effect on removal performance. The increase in Ni and V removal with SZ loading (SZ) is explained by the greater number of active sites available over the adsorbent^32^.

Removal efficiencies were interpreted by Konne et al.^31^ that reported, at 2 g. of SZ, equilibrium was easily reached, and that adding more adsorbent (SZ) crowded some of the available sites, decreasing rather than increasing adsorption potential.

##### Effect of initial metal ions concentration (Cr^2+^, Ni^2+^, V^4+^ and Zn^2+^) (BD crude oil quantity)

**Fig. 19:**
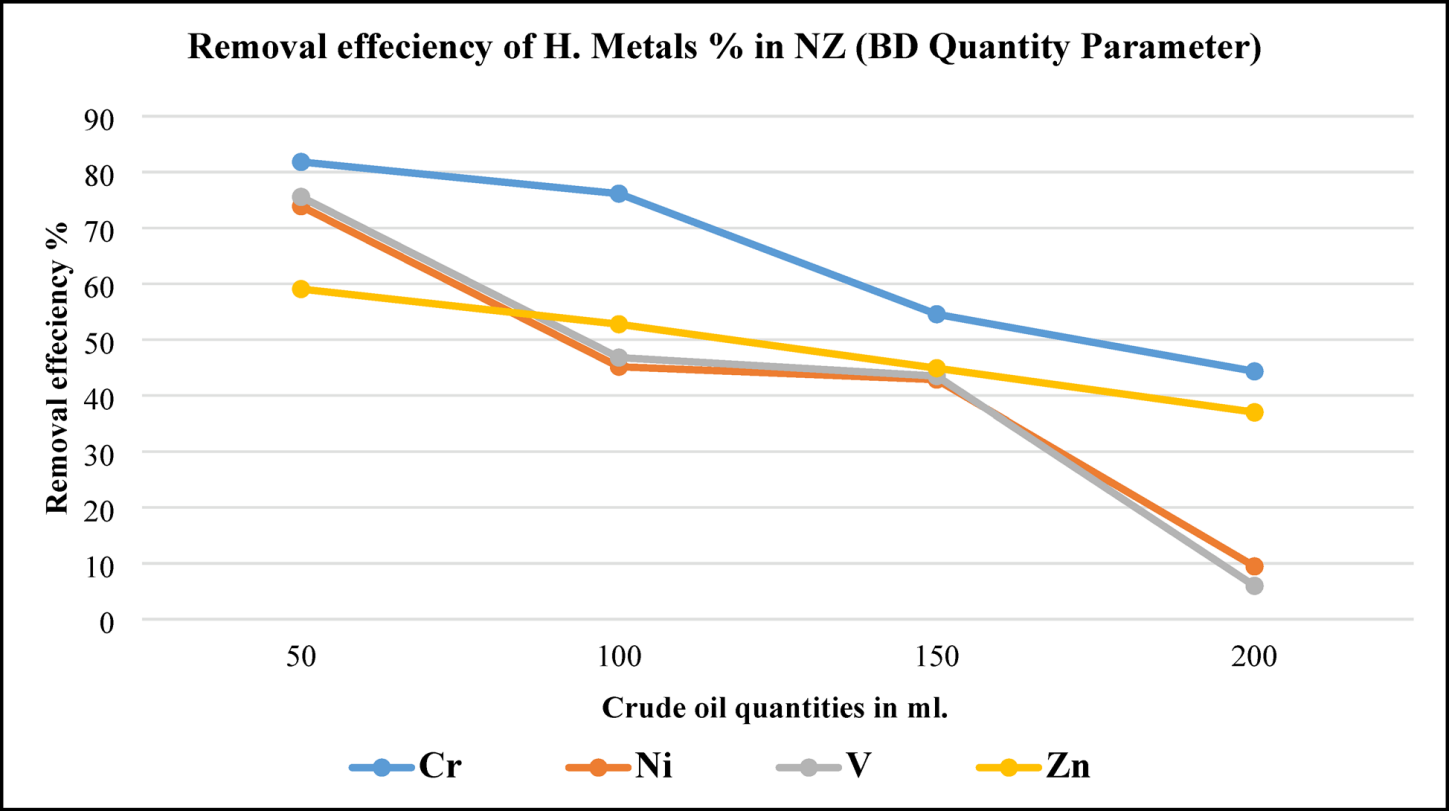
Metals removal efficiency from BD as a function of BD quantity using NZ.

Figure 19, illustrates (Cr, Ni, V, and Zn) metals removal efficiency as a function of crude oil quantity using NZ. Maximum removing efficiency has occurred at minimum BD quantity (50 ml.) and efficiency gradually decreased to minimum values at maximum BD quantity (200 ml.). It was observed that there was by increasing crude oil (BD) quantities a rapid remarkable decrease in removing efficiency for all studied metals. That may be referred to as the adsorbent (NZ) active sites will be quickly saturated with metal ions and that as equilibrium is achieved, adding more crude oil simply raises the analytic concentration. Results agree with results were obtained before by^31^.

**Fig. 20:**
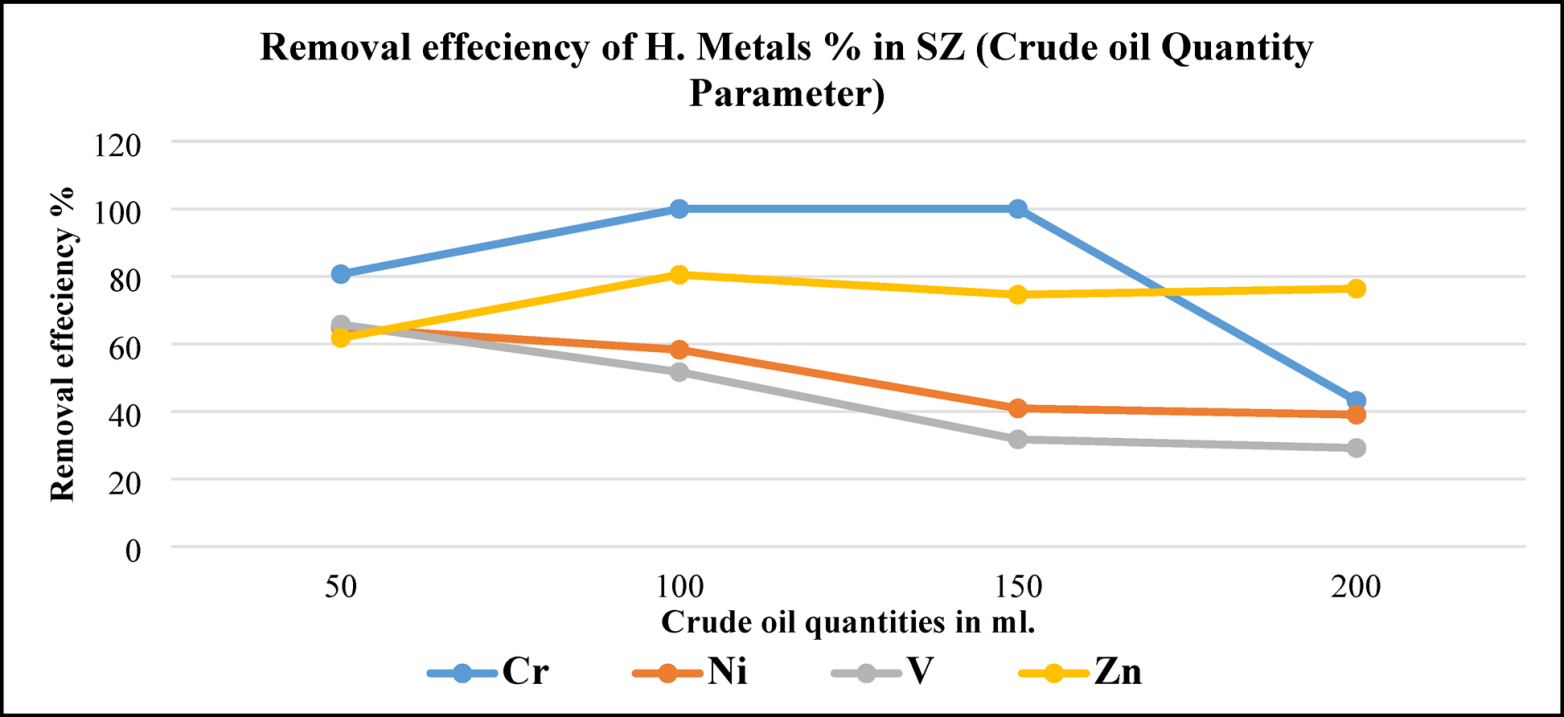
Metals removal efficiency from BD at different crude oil quantities using SZ.

Figure 20, shows metals removal efficiency by using different BD quantities using SZ (0.5 g.) for 18 h. contact time. Cr removing efficiency increased from 80% by using 50 ml. BD to 100% (Cr completely extracted) by using 100- and 150-ml. BD the dramatically decreasing in Cr removing efficiency by using 200 ml. of BD. While Ni and V removing efficiency took the same behavior for removing efficiencies, maximum efficiency by using 50 ml. BD and decreased gradually to minimum efficiencies by using 200 ml. of BD. Zn removing efficiency was increased from 62 to 80% by using 50 to 100 ml. of BD then decreased slightly by using 150 and 200 ml. of BD. Generally, it was observed that by increasing crude oil quantities, metal removal efficiencies were decreased also. These results agree with results were obtained before by^31^.

## Conclusion

In the present work, Crude oil [Belayim Desert crude oil (BD)] was received from Belayim oil field, Egypt was characterized as fingerprinting techniques used for crude oil characterization, the results concluded, API classified BD is a heavy crude oil type. High heavy metals content occurred in BD that abundantly with V > Ni > Zn > Cr. Two different types of zeolites (Natural and synthetic) were applied in Demetallization process for BD. Studied zeolites NZ and SZ were characterized using ICP-OES, XRD analysis and SEM micrographs moreover EDX analysis.

This study examined the impacts of several operating factors on the effectiveness of removing metals from BD crude oil, including contact time, zeolites load, and crude oil quantities. Findings showed that the following:

### NZ investigations

The optimal removal conditions using NZ occurred after 6 h. contact time, when using 0.5 g. of NZ stirred with 50 ml crude oil, these experimental conditions produce maximum V, Ni, and Cr removal efficiencies as 75.6, 73.9, and 81.8% respectively, but NOT maximum Zn removal efficiency reached 59% only. PXRD & EDX analysis emphasis the highly tendency of NZ to adsorb metallic ions from BD crude oil.

### SZ investigations

The optimal removal conditions using SZ occurred after 12 h. contact time, when using 2 g. of SZ stirred with 50 ml crude oil, these experimental conditions produce maximum Zn and Cr removal efficiencies as 70.5, 69.17% respectively, but NOT maximum V and Ni removal efficiency reached to maximum after 26 h. contact time, 2 g. SZ with 50 ml. crude oil 68.9%.

The synthetic zeolite (SZ) under investigation has demonstrated a beneficial effect on metal removal.

From the current study, general conclusions could be obtained as follows:

- Natural Zeolite (NZ) is more efficient than Synthetic Zeolite (SZ) in extracting Ni and V metals in BD crude oil.
- The selectivity series of NZ for heavy metals as per metals removal efficiencies are Cr > V > Ni > Zn.
- The selectivity series of SZ for heavy metals as per metals removal efficiencies are Cr > Zn > V > Ni.

Also, All Experimental results of adsorption test showed that Langmuir isotherm predicts well the experimental data. RDX and EDX analyses confirm the high tendency of NZ greater than SZ to remove Ni, V metal ions from crude oil (specific type BD). Long term tests revealed the high stability of NZ for Ni and V removal.

## Data Availability

The datasets used and/or analysed during the current study available from the corresponding author on reasonable request.
